# Paracrine effects of intraocularly implanted cells on degenerating retinas in mice

**DOI:** 10.1186/s13287-020-01651-5

**Published:** 2020-03-31

**Authors:** Xiao Liu, Fenghua Chen, Yao Chen, Huayi Lu, Xiaoqin Lu, Xiaoyan Peng, Henry J. Kaplan, Douglas C. Dean, Ling Gao, Yongqing Liu

**Affiliations:** 1 Department of Ophthalmology and Visual Sciences, University of Louisville School of Medicine, 301 E Muhammad Ali Blvd, Louisville, KY 40202 USA; 2 Department of Ophthalmology, Second Xiangya Hospital of Central South University, Changsha, China; 3 Department of Ophthalmology, Beijing Tongren Hospital, Capital Medical University, Beijing, China; 4 Department of Ophthalmology, Xiangya Hospital of Central South University, Changsha, China; 5 Second Hospital of Jilin University, Changchun, Jilin Province China; 6 James Graham Brown Cancer Center, Louisville, USA; 7 Birth Defects Center, University of Louisville School of Medicine, Louisville, KY USA

**Keywords:** Cell-based therapy, Retinal degeneration, Paracrine effect, Intraocular transplantation

## Abstract

**Background:**

Retinal degeneration is a leading cause of blindness in the world; its etiology is complex and involves genetic defects and stress-associated aging. In addition to gene therapies for known genetically defective retinal degeneration, cellular therapies have been widely explored for restoring vision in both preclinical animal models and clinical trials. Stem cells of distinct tissue sources and their derived lineages have been tested for treating retinal degeneration; most of them were reported to be effective to some extent in restoring/improving deteriorated vision. Whether this visual improvement is due to a functional integration of grafted cells to substitute for lost retinal neurons in recipients or due to their neuroprotective and neurotrophic effects to retain recipient functional neurons, or both, is still under debate.

**Methods:**

We compared the results of subretinal transplantation of various somatic cell types, such as stem cells and differentiated cells, into Rho^P23H/+^ mice, a retinal degeneration model for human *retinitis pigmentosa* (RP) by evaluating their optokinetic response (OKR) and retinal histology. We identified some paracrine factors in the media that cultured cells secreted by western blotting (WB) and functionally evaluated the vascular endothelial growth factor Vegfa for its potential neurotrophic and neuroprotective effects on the neuroretina of model animals by intravitreal injection of VEGF antibody.

**Results:**

We found that live cells, regardless of whether they were stem cells or differentiated cell types, had a positive effect on improving degenerating retinas after subretinal transplantation; the efficacy depended on their survival duration in the host tissue. A few paracrine factors were identified in cell culture media; Vegfa was the most relevant neurotrophic and neuroprotective factor identified by our experiments to extend neuron survival duration in vivo.

**Conclusions:**

Cellular therapy-produced benefits for remediating retinal degeneration are mostly, if not completely, due to a paracrine effect of implanted cells on the remaining host retinal neurons.

## Background

Age-related macular degeneration (AMD), *retinitis pigmentosa* (RP), diabetic retinopathy (DR), and glaucoma-induced degeneration of retinal ganglion neurons are the major retinal disorders and leading causes for blindness worldwide. Their etiologies are distinct and complex and involve genetic defects and stress-associated aging [1, 2]. Their chronic progression leads to the impairment and even loss of vision [3]. A complete cure for these retinal disorders is very challenging, although advanced gene therapies for certain genetic defect-caused RP have been successfully practiced in the clinic [4, 5]. Stem cell-based therapies are basically targeting the replacement of lost and diseased retinal neurons and retinal pigment epithelium (RPE) cells and have demonstrated their potential in restoring the deteriorated vision in both model animals and clinical trials [2, 6, 7]. However, whether this visual restoration is due to a functional integration of the grafted cells to substitute for lost retinal neurons in recipients or due to their neuroprotective and neurotrophic effects to retain recipient functional neurons, or both, is still under debate. In general, pluripotent stem cells (PSCs), such as embryonic stem cells (ESCs) and induced pluripotent stem cells (iPSCs), must first be differentiated in vitro into a target cell type, such as photoreceptors (PRs), RPE cells, or retinal ganglion cells (RGCs), prior to transplantation to recipients [1, 8]. In contrast, adult stem cells, such as bone marrow-derived stromal cells (BMSCs), adipose stem cells (ASCs), retinal stem cells (RSCs), and umbilical cord stem cells (UCSCs), can be directly grafted to the diseased eyes to remediate their deteriorating vision [1, 9–12]. It is speculated that PSC-derived target cells restore vision mainly by cell substitution, whereas adult stem cells would rescue vision essentially by paracrine effects because no cell substitution was observed in the grafted eyes [1, 2, 6, 13].

No direct comparison of the effectiveness has been made between the abovementioned two strategies, i.e., PSCs vs. adult stem cells, though more and more BMSCs were used to treat model animals and in clinical trials because of their autologous nature, abundance, and convenience [1]. It seems that using adult stem cells to treat retinal degeneration disorders has more advantages over using PSCs [14]. However, no experiment has ever been performed to test whether differentiated somatic cells, particularly those ocular cells, can also be as effective as stem cells or even better. We therefore sought to systematically compare these two cell types in terms of improving the deteriorated vision in model animals and to explore the underlying mechanism(s). Here, we provide evidence that RPE sphere-derived stem cells (SDSCs) prepared from mouse RPE cells could integrate into the grafted retinas and restore the decreased vision of Rho^P23H/+^ mice after subretinal transplantation. Similarly, the subretinal transplantation of several mouse primary cultured cells including mouse embryonic fibroblasts (MEFs), Müller glial cells (MGCs), and RPE cells also displayed such a capacity to improve their vision by retaining a significant number of photoreceptors compared to PBS-sham-treated controls, although minimum cell migration and tissue integration were observed. In agreement with the microarray data, we detected secretory factors/cytokines in cell culture supernatants. We conclude that both stem cells and differentiated cells can nurture and protect degenerating retinas from further degeneration by secreting neurotrophic and neuroprotective factors, among which the vascular endothelial growth factor Vegfa is the major effecter in protecting and prolonging retinal neurons.

## Materials and methods

### Animals

B6.129S6(Cg)-*Rho*^*tm1*.*1Kpal*^/J transgenic mice, with defective mouse rhodopsin at amino acid sequence site 23 where proline (P) is changed to histidine (H) by a knock-in insertion [15, 16], were purchased from The Jackson Laboratory (stock # 017628). We maintained them by breeding within siblings and bred them with C57BL/6J for Rho^P23H/+^ in our animal facility at Louisville. The homozygous mutant P23H mice (Rho^P23H/P23H^) were bred to wild-type B6 mice to produce heterozygous offspring (Rho^P23H/+^) for cell transplantation experiments as recipients with retinal degeneration. Rho^P23H/+^ mice were born with normal vision, but their retinas started to degenerate, and their vision decreased around postnatal day (P) 40. By approximately P60, they lost almost 1/3 of photoreceptor cells in the outer nuclear layer (ONL) and had little optokinetic response. At approximately P40, one eye of these Rho^P23H/+^ mice subretinally received an injection of 2 μl of 1 × 10^5^ cultured cells, whereas the other eye received the same amount of PBS as a negative control. For anti-VEGF treatment, both eyes of Rho^P23H/+^ and Rho^+/+^ mice at P40 received an intravitreal injection of 75 μg/eye anti-VEGF antibody (3 μl of bevacizumab at the concentration of 1.25 mg/0.05 ml) or the same amount of PBS as a negative control. Animal breeding, husbandry, and surgery procedures were in accordance with the Association for Research in Vision and Ophthalmology (ARVO) Statement for the Use of Animals in Ophthalmic and Vision Research and were approved by the University of Louisville Institutional Animal Care and Use Committee (IACUC) regulation.

### Cell preparation

We purchased from the Jackson Laboratory both Cre-tdTomato mice (B6.Cg-Gt (ROSA)26Sor^tm9(CAG-tdTomato)Hze^/J, stock # 007909) that have a *loxP*-flanked STOP cassette preventing transcription of a CAG promoter-driven red fluorescent protein variant (tdTomato) and BEST1-Cre mice (C57BL/6-Tg (BEST1-cre)1Jdun/J, stock # 017557) that express Cre recombinase under the control of the human BEST1 promoter. When both mice are crossed, the RPE cell-specific BEST1 promoter activates the expression of Cre recombinase that specifically removes *loxP*-flanked STOP cassette resulting in the expression of tdTomato red fluorescence to permanently tag the RPE cells no matter whether their identity has been changed or not. Similarly, mouse embryonic fibroblasts (MEFs) were isolated as previously described [17], from E19.5 *S100a4*-tdTomato embryos generated by a cross between the Cre-tdTomato mice with the S100a4-Cre mice (B6.C-Tg(S100a4-cre)1Egn/JhrsJ, stock # 030644) that express Cre recombinase under the control of the promoter of human S100a4, a fibroblast-specific protein (FSP1), whereas mouse Müller glial cells (MGCs) and retinal pigment epithelium (RPE) cells were isolated from P20 *Pdgfra*-tdTomato or the above *BEST1*-tdTomato pups, respectively, as previously described [18]. The isolated MEFs, MGCs, and RPEs were all lineage-specifically tagged with tdTomato red fluorescence. Unlike RPEs and MGCs, MEFs were initially highly heterogenous right after isolation from the mixed embryonic tissues. However, after multiple passages (> P10), most MEF cells, if not 100%, were pure tdTomato positive fibroblasts because they have the best proliferation rate among the initial heterogenous populations. We also created an immortalized sphere-derived stem cell (SDSC) line from the abovementioned RPE cells. Cells were maintained in Dulbecco’s modified Eagle’s medium (DMEM) with 10% fetal bovine serum (FBS), passaged 2–3 times, and individualized by 0.25% trypsin and resuspended at a concentration of 1 × 10^5^/μl in PBS for transplantation.

### Subretinal injection

For subretinal transplantation of cells, the heterozygous Rho^P23H/+^ mice at P40 were anesthetized by an intraperitoneal (IP) injection of a mixture of 100 mg of ketamine and 10 mg of xylazine per kg of body weight, and then a mydriatic eye drop was given to dilate the pupil. A tiny bleb was visible below the retina on the superior nasal location by injecting 2 μl of 1 × 10^5^ cells through the sclera approximately 1 mm behind the limbus under a surgical microscope using a 30-gauge blunt needle and a 5-μl syringe as previously described [19].

### Visual optokinetic response (OKR) assessment

Visual function was assessed using a noninvasive OptoMotry© optokinetic testing system (CerebralMechanics). A test mouse was placed unrestrainedly on the central platform after a 30-min dark adaptation and surrounded by 4 monitors displaying alternative black and white vertical bars. Her reflexive head movement behavior was recorded by two independent observers blinded to treatments as previously reported [20].

### Neural differentiation of SDSCs

Spheres were allowed to form by dissociating monolayer SDSCs into small clumps with 1 mg/ml type IV collagenase and cultured in 60-mm ultralow adhesion plates in DMEM with 10% FBS for 3 days. The spheres were then transferred to a glass chamber slide coated with 0.1% gelatin and cultured for 18 days in neural differentiation medium containing DMEM/F12, 10% knockout serum replacer, neuronal culture supplements N2 and B27 (Invitrogen), 1 ng/ml DKK1 (R&D), 1 ng/ml noggin (R&D), and 1 ng/ml IGF1 (R&D). The medium was refreshed every other day.

### Immunofluorescence (IF)

Cells cultured in 8-well chamber glass slides coated with 0.1% gelatin were fixed with 4% paraformaldehyde, rinsed with PBS, and blocked with 1% bovine serum albumin (BSA) and 3% serum isolated from the species where the primary antibody was raised. The primary and secondary antibodies used for IF are listed in Supplementary Table S1. Nuclei were counterstained with Hoechst dye (Invitrogen, Cat. # H1399, 1:500), and images were captured by an inverted fluorescence microscope.

### Immunohistochemistry (IHC)

Upon euthanization, both cell-transplanted and PBS-sham-treated eyes were enucleated and immediately immersed in CO_2_-independent media on ice. Eyeballs for cryosection were fixed in 4% paraformaldehyde in 0.1 M PBS for 20 min, followed by three washes of PBS and thereafter cryoprotected through 5%, 10%, and 15% sucrose for 1 h sequentially and 20% sucrose overnight. The cryoprotected eyes were finally embedded in optimal cutting temperature (OCT) compound with 20% sucrose for 30 min and then cryosectioned at 10 μm. The paraffin-embedded sections of eye tissues were deparaffinized by xylene and rehydrated with a series of ethanol solutions and into a final solution of PBS. Tissues were sectioned at 5 μm and H&E stained. Sections were blocked with 2% BSA, 5% serum, and 0.1% Triton X-100 at 25 °C for 1 h and then incubated first with primary antibodies and then with secondary antibodies (Supplementary Table 1). Nuclear staining and photography were conducted as with IF.

### Affymetrix microarray analyses

Total RNA of SDSCs was extracted using TRIzol solution (Invitrogen) followed by RNeasy column cleanup (QIAGEN) according to the manufacturer’s instructions. Probes were prepared for hybridization to the mouse genome 430 2.0 GeneChips according to the manufacturer’s instructions (Affymetrix). The hybridization images were processed using Affymetrix GeneChip Command Console® (AGCC) software [17]. Microarray datasets for MEFs, MGCs, and RPE cells in the same Affymetrix platform were downloaded from the public Gene Expression Omnibus (GEO) database, which includes three biological samples for each cell type (GSM1712851, GSM1712852, and GSM1712853 for MEFs; GSM671985, GSM671986, and GSM671987 for MGCs; GSM1291057, GSM1291058, and GSM1291059 for RPE cells). The raw data of intensities were log2-transformed and normalized across all samples and gene spots on a GC content background correction. The final expression level of each gene was set as relative to that of the housekeeping gene *Actb* so that comparisons can be made between samples and between genes.

### Real-time quantitative PCR (qPCR)

Total RNA was isolated using TRIzol solution (Invitrogen), and cDNAs were prepared using an RT kit (Invitrogen) according to the manufacturer’s instructions. Primer sets for the selected genes were designed by the online program “Primer3.” Sequences of the primers are listed in Supplementary Table S2. PCRs were performed with the Stratagene real-time PCR system 3000P, and cycle threshold (Ct) numbers of each gene were collected and analyzed using the double delta formula [21]. The expression of each gene was normalized to that of the housekeeping gene *Actb* or *Gapdh*. Three biological replicates with two technical replicates were performed on each cell sample.

### Protein sample preparation

Total cell soluble proteins were prepared as described previously [22]. The trichloroacetic acid (TCA)-deoxycholate (DOC) protein precipitation method was used to concentrate proteins in cell culture-conditioned media as previously reported [23]. Briefly, three 10-cm plates of adherent cells were cultured in DMEM with FBS until confluence, when the medium was replaced with fresh medium without FBS for an additional 1 day. Cell-conditioned media were collected and centrifuged at 300×*g* for 10 min and either stored at − 80 °C or immediately used. Then, 1% (v/v) of 2% sodium deoxycholate solution and TCA were added to each tube at a final concentration of 7.5% (v/v) and left on ice. Proteins were precipitated by centrifugation at 15,000×*g* for 20 min at 4 °C, and the supernatants were discarded. Ice-cold (− 20 °C) acetone was added to the pellets at the ratio of 1:2, gently vortexed, and kept at − 20 °C. After centrifugation at 15,000×*g* for 5 min at 4 °C, the pellets were rinsed twice with ice-cold (− 20 °C) acetone and then centrifugation at 15,000×*g*. The pellets were air-dried and dissolved in 210 μl of extraction buffer (7 M urea, 2 M thiourea, 30 mM TRIS, and 4% CHAPS detergent, pH 8.5) and kept at − 80 °C until use.

### Western blotting (WB)

Ten micrograms of the above crude protein lysate was mixed with 1 μl of loading dye buffer and directly loaded onto a 4–21% gradient SDS-PAGE gel at 95 °C for 1 min together with prestained protein molecular weight markers. The electrophoresis was run at a constant voltage of 120 V until the front line was close to the edge of the gel. Gel proteins were then transferred to a PVDF membrane at 4 °C overnight. The protein blot was initially stained with 0.5% Ponceau S to indicate total amounts of proteins on the membrane; thereafter, it was used for hybridization with the primary antibodies listed in Supplementary Table 1. Blocking, primary and secondary antibody incubations, and the enhanced chemical luminescence (ECL) process were performed as previously described [22].

### Statistical analysis

Comparisons between cell lines were assessed using the two-tailed and unpaired Student’s *t* tests. All values in the graphs are presented as the means ± standard deviations. Three stars “***” indicate a *p* value ≤ 0.001, two stars “**” indicate a *p* value ≤ 0.01, and one star “*” indicates a *p* value ≤ 0.05. For in vitro studies including qPCR and cell growth rate calculations, the results were obtained from at least 3 independent experiments of 3 technical replicates or as otherwise specified.

## Results

### Sphere-derived stem cells (SDSCs) can differentiate into cells expressing neuroretinal markers

**Fig. 1:**
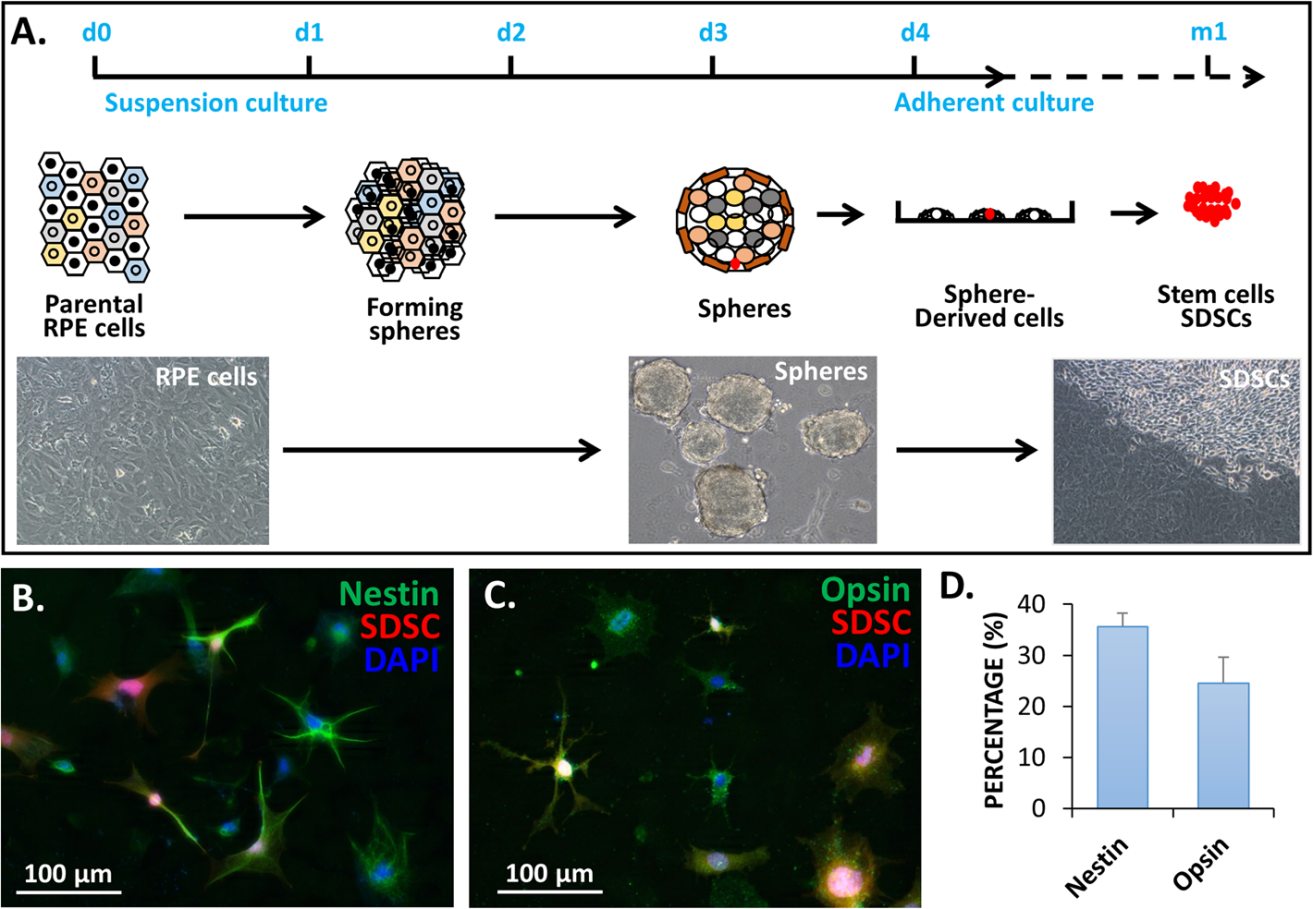
RPE cell sphere-derived stem cells (SDSCs) and their neural differentiation in vitro. **a** A schematic diagram showing SDSC production. RPE cells were adherently cultured to confluence, and the monolayer cultured cell sheet was scraped off the plate and resuspended in an ultralow culture dish (d0) where the cells initially formed aggregates and then spheres in 3 days (d3). These 3-day spheres were placed back into a culture plate coated with gelatin for up to a month to observe stem cell colony formation (see [17] for the detailed procedure). Different colors indicate the heterogeneous RPE-derived cell populations. **b** SDSCs were cultured in a photoreceptor differentiation medium for 20 days and then immunostained with the general neural cell marker nestin and **c** the rod photoreceptor marker opsin. **d** Their differentiation rates in the expression of nestin and opsin

Using our sphere-induced reprogramming technology, we created SDSCs from *BEST1*-tdTomato RPE cells (Fig. 1a), which manifested stem cell properties that included both self-renewal and multipotential capacities as reported previously [17]. As reported with other bone marrow-derived stromal cells (BMSCs) [24], SDSCs could also differentiate into neuron-like cells after being cultured in a neural differentiation medium for 3 weeks. They expressed both the neural progenitor marker nestin and the photoreceptor cell marker opsin, and their neural differentiation rate was approximately 30% (Fig. 1b–d).

### Rho^P23H/+^ mice start to reduce photoreceptor cells and the optokinetic response around P40

**Fig. 2:**
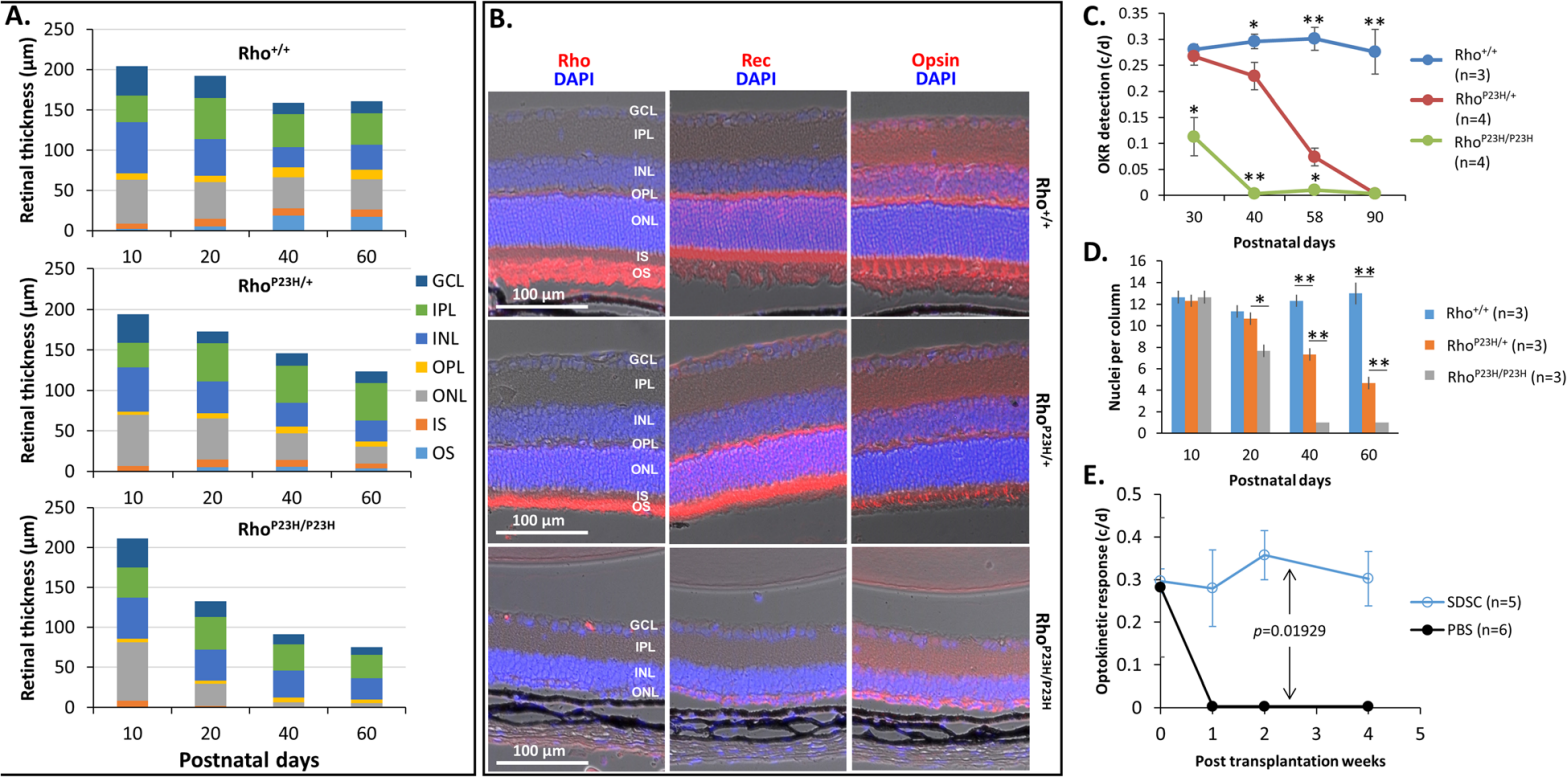
Transplantation of SDSCs in Rho^P23H/+^ recipients. **a** Comparison of retinal thickness between mutant (homozygous Rho^P23H/P23H^ and heterozygous Rho^P23H/+^) and wild-type mice (Rho^+/+^) in stack bar graphs. **b** Representative P40 retinal cross paraffin sections of the three genotypes stained with the rod markers rhodopsin (Rho for the outer segment) and recoverin (Rec for the inner segment) and the cone marker opsin. **c** The optokinetic response (OKR) of the three different genotypes of mice at postnatal days 30 to 90. **d** Number of nuclei per column in the outer nuclear layer. **e** Vision assessment by an OKR system for Rho^P23H/+^ mice either subretinally injected with 2 μl of 2 × 10^5^ SDSCs or PBS as a control. GCL, ganglion cell layer; IPL, inner plexiform layer; INL, inner nuclear layer; OPL, outer plexiform layer; ONL, outer nuclear layer; IS, inner segment; OS, outer segment; c/d, cycles/degree

Rho^P23H/+^ mice are a mouse model for human *retinitis pigmentosa* (RP), where a human P23H mutated rhodopsin gene *Rho* for the autosomal dominant RP is knocked in [16]. Sakami et al. reported that by P35, nine tenths of photoreceptor cells in the outer nuclear layer (ONL) in homozygous Rho^P23H/P23H^ mice were lost, while only approximately one third of them were degenerated in heterozygous Rho^P23H/+^ mice [16]. We found that unlike wild-type Rho^+/+^ mice whose retinal thickness was stabilized after P40, both Rho^P23H/P23H^ and Rho^P23H/+^ mutant mice showed a continuous shrinkage of retina thickness. The reduction of retinal thickness was quicker in Rho^P23H/P23H^ than in Rho^P23H/+^ mice (Fig. 2a). The major reduction fraction of the retinal thickness was the ONL and the Rho^+^ outer segment (OS) (Fig. 2a–b), resulting from an actual loss of photoreceptor cells when only one single nuclear layer in the ONL was detected at P40 in homozygous Rho^P23H/P23H^ mice (Fig. 2a–b). As a result, Rho^P23H/P23H^ and Rho^P23H/+^ mice became blind at P40 and P90, respectively, based on their optokinetic response (OKR) (Fig. 2c). In our pre-experimental test, we subretinally injected SDSCs into Rho^P23H/P23H^ mice at P30 when 1/2 of their vision was lost (Fig. 2c), and we found that the OKR values of the mice with the transplanted cells did not show any improvement in visual response compared to the PBS-controlled mice (data not shown), suggesting that transplantation of cells to the Rho^P23H/P23H^ mice to rescue their degenerating vision might be too late at P30 when the retinal degeneration was accelerated. Apparently, Rho^P23H/+^ mice would provide a much-needed broad window for our cell transplant experiments below.

### Subretinal transplantation of SDSCs retains the vision of Rho^P23H/+^ mice

To check whether SDSCs would rescue the deteriorated vision of retinal degeneration animals, a total of 2 × 10^5^ individual SDSCs were delivered to the subretinal space in Rho^P23H/+^ mice at P40 when 1/3–1/4 of their photoreceptor cells were lost and their vision started to decrease compared to the wild-type Rho^+/+^ mice (Fig. 2a–d) [15]. Within 4 weeks post cell transplantation at P40, the visual acuity assessed by the OKR showed that all PBS-sham-treated control eyes had little response (< 0.01), whereas those eyes with the transplanted SDSCs still maintained the prior levels (Fig. 2e), suggesting that SDSCs have the ability to rescue the degenerating vision of Rho^P23H/+^ mice.

### Integration of the transplanted SDSCs in Rho^P23H/+^ retinas

**Fig. 3:**
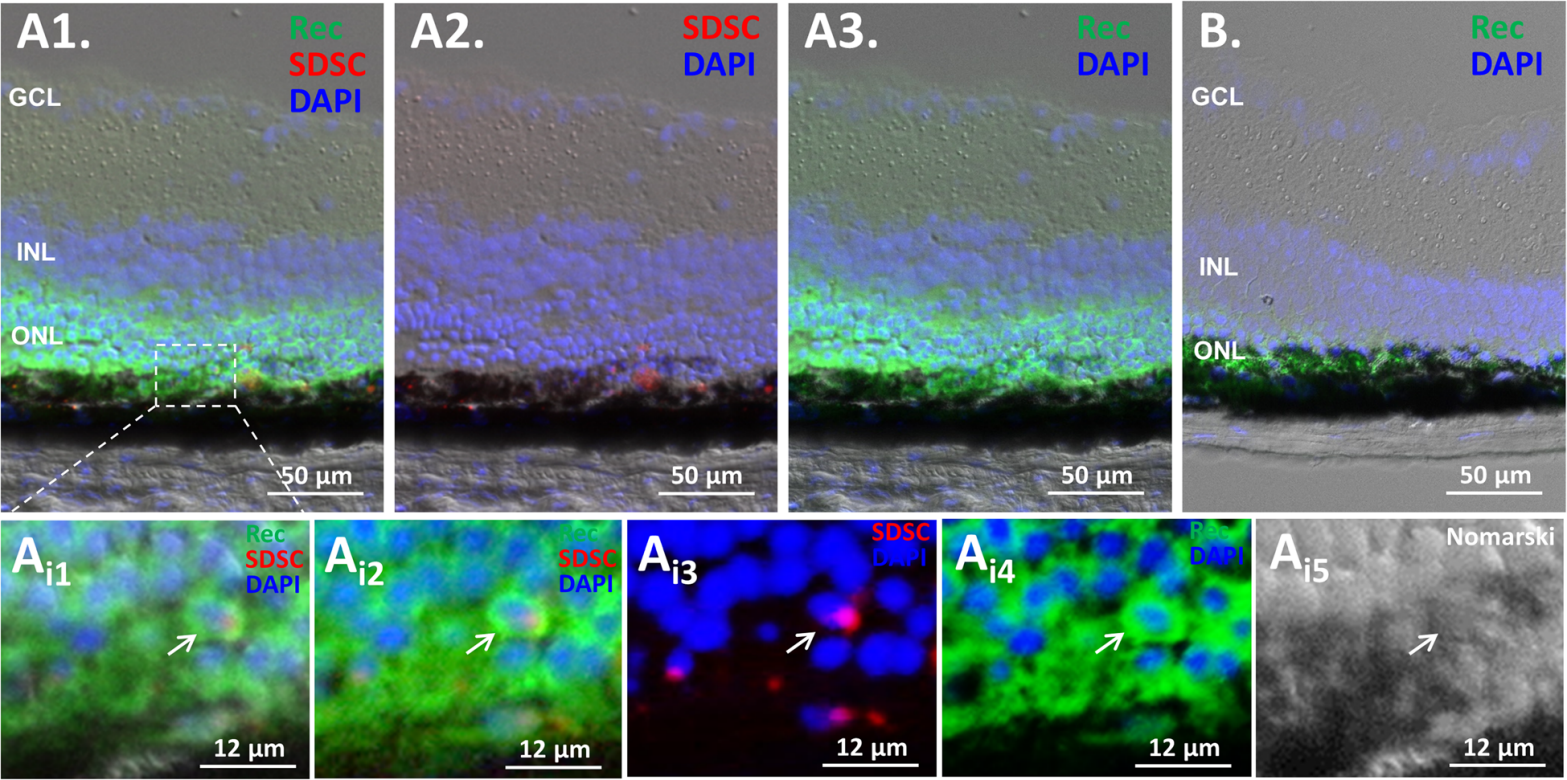
Subretinal transplantation of SDSCs to Rho^P23H/+^ mice. **A** Twenty-eight days after subretinal transplant, most of the grafted SDSCs with red fluorescent tdTomato expression were retained in the subretinal space, while some of them moved and possibly integrated into the ONL and expressed the photoreceptor marker recoverin (Rec) on a representative frozen section. Note that more nuclei were retained in the ONL compared to **B** the PBS control retina. Insert is an enlargement of the defined area to indicate the integration of a grafted SDSC expressing Rec (white arrow). GCL, ganglion cell layer; INL, inner nuclear layer; ONL, outer nuclear layer

**Fig. 4:**
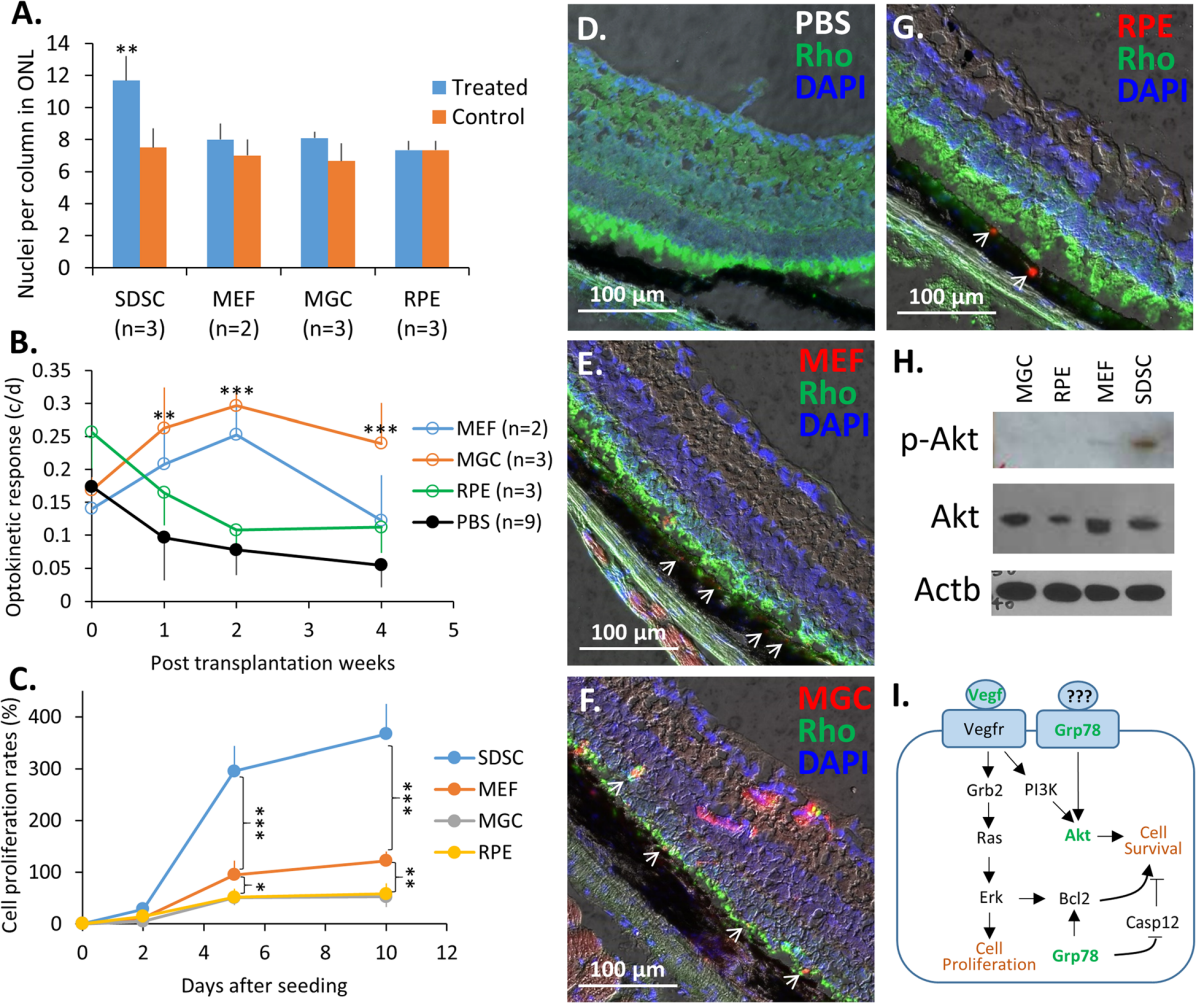
Effects of subretinally transplanted cells on retinal structure and vision of the Rho^P23H/+^ mice. **a** Comparisons of the number of nuclei per column in the ONL between cell-transplanted retinas (treated) and PBS controls. **b** Optokinetic responses of the retina transplanted with different primary cells over the next 4 weeks after transplantation. The paired two-tail Student’s *t* test was performed and statistical significance was marked for different cell transplantations compared to the pooled PBS control. **c** Proliferation rates of different cell types in culture. A representative image of frozen sections of retinas grafted with **d** PBS control, **e** MEFs, **f** MGCs, and **g** RPE cells. **h** Activation of the PI3K/Akt pathway was detected by WB in SDSCs. SDSCs, sphere-derived stem cells; MEFs, mouse embryonic fibroblasts; MGCs, Müller glial cells; RPEs, retinal pigment epithelial cells; p-Akt, phosphor-Akt at site Ser473. **i** A diagram depicting two possible signaling pathways through activation of Akt, leading to the survival of the remaining photoreceptor cell of the degenerating retina and/or the transplanted cells. “???” indicates an unknown ligand(s) and the green-colored proteins were investigated in this study. Statistically significant at *p < 0.05, **p < 0.01, and ***p < 0.001. White arrows indicate possible transplanted cells

To check if the transplanted SDSCs could rescue degenerating retinas, we immunostained the cryosections of transplanted eyes with the photoreceptor marker recoverin (Rec) and found that more photoreceptors were retained compared to PBS-sham-treated control eyes at 28 days posttransplantation (Figs. 3A, B and 4a). More transplanted cells were located around the injection sites, and a few of them appeared to have differentiated to express the photoreceptor marker Rec in the recipient ONL whether these double-stained cells were truly fate-switched transplanted cells or they were fussed with local recipient photoreceptor cells remains unclarified at this moment (Fig. 3A_i_, white arrow). H&E-stained paraffin sections confirmed that more photoreceptors in the ONL were retained in the SDSC-transplanted eyes (Fig. 4a). Consequently, these cell-transplanted eyes manifested a much better vision than the PBS control eyes (Fig. 2e). It appeared that the mechanism for transplanted SDSCs to rescue degenerating retinas was not through the replacement of lost retinal neurons because too few SDSCs were integrated into recipient retinas (Fig. 3A). It is therefore likely that through a paracrine effect of transplanted SDSCs, the remaining retinal neurons are protected.

### Implantation of primary differentiated cells also delays the vision loss of Rho^P23H/+^ mice

As the capacity of SDSCs to differentiate into retinal neurons to functionally replace lost neurons was seemingly not required for them to rescue the degenerating vision of Rho^P23H/+^ mice, we reasoned that other differentiated cells might also possess such an ability to remediate retinal degeneration. To test this hypothesis, we subretinally delivered 2 × 10^5^ cells of three different lineages, i.e., mouse embryonic fibroblasts (MEFs), Müller glial cells (MGCs), retinal pigment epithelial (RPE) cells, and the same volume of PBS as a sham-treated control, into Rho^P23H/+^ mice at P40 and assessed their OKR over the next 4 weeks. As speculated, transplanted cells improved the vision of grafted eyes to various degrees in Rho^P23H/+^ mice compared to that of PBS-sham-treated controls (Fig. 4b). MEFs and MGCs showed a better OKR than RPE cells, although MGCs and RPE cells are adult-differentiated cells with a limited proliferative capacity, whereas MEFs are of embryonic origin with a higher proliferative capacity (Fig. 4c). The reduction in OKR was reflected by the loss of photoreceptor cells revealed by H&E staining. Compared to PBS control (Fig. 4d), eyes grafted with either MEFs (Fig. 4e) or MGCs (Fig. 4f) retained more photoreceptors, whereas no difference was detected between RPE cell-transplanted eyes and their control eyes (Fig. 4a). Taken together, it appeared that the degree to which different primary cells improve the degenerating vision of grafted eyes was not dependent on whether they were ocular (i.e., MGC and RPE) or nonocular (i.e., MEF) sources but was likely associated with their survival duration and capacity to secrete paracrine factors to nurture and/or to protect remaining retinal neurons.

### Survival of transplanted cells in recipients

Compared to SDSCs (Fig. 3), 4 weeks after transplantation, much fewer differentiated cells were observed at injection sites (Fig. 4e–g). With the exception of MEFs that seemingly stayed in the subretinal space (Fig. 4e), a few MGCs were spotted in the retina (Fig. 4f), while a few RPE cells were detected in the RPE (Fig. 4g). It appeared that both cultured MGCs and RPE cells could find a path to their compatible tissue [25, 26]. However, the migration of transplanted cells to their compatible tissues was seemingly not a functional integration because it did not correlate with the gain of ONL cells and visual function (Fig. 4a, b). Because the PI3K/Akt pathway is important for cell survival, we reason that phosphorylation of Akt-Ser473 would facilitate cell survival. Indeed, phosphorylation of Akt-Ser473, as detected by western blotting (WB), was higher in SDSCs, lower in MEFs, and absent in both RPE cells and MGCs (Fig. 4h), suggesting that SDSCs had a higher survival capacity than the rest of the cells and thereby might live longer in host eyes to secrete more paracrine factors to support photoreceptor function.

### Expression of neurotrophic and neuroprotective genes in ocular and nonocular cells

**Fig. 5:**
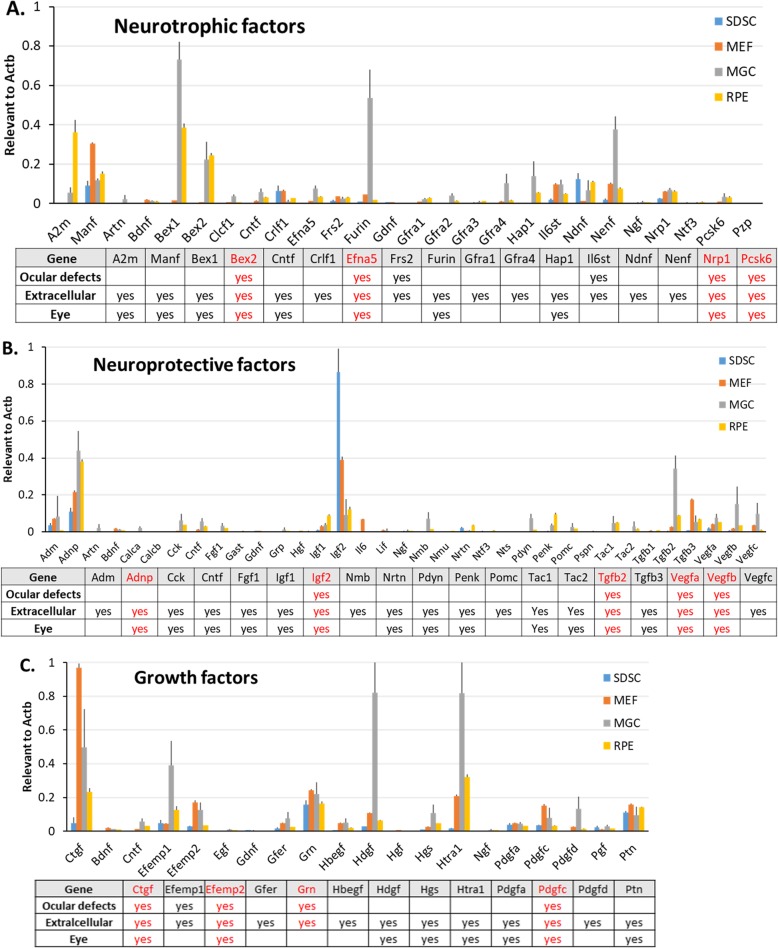
Preidentification of neurotrophic, neuroprotective, and growth factors for remediation of retinal degeneration. Selections were made based on Affymetrix microarray gene expression data plus information on their ocular defect by gene mutation and their expression location. Red-colored genes were selected for further qPCR-based gene expression confirmation. Bar graphs of selected **a** neurotrophic factors, **b** neuroprotective factors, and **c** growth factors

To explore the molecular mechanism(s) underlying the positive effects of transplanted cells on the remediation of retinal degeneration, we sought to analyze gene expression data from the microarray experiments that we either performed for SDSCs or downloaded for MEFs, MGCs, and RPE cells from the public gene expression omnibus (GEO) database of the National Center for Biotechnology Information (NCBI). We focused on those neurotrophic and neuroprotective genes that encode extracellular (secretory) factors defined by Mouse Genome Informatics (MGI) and selected from the MGI database using the keywords “extracellular factor” plus “neurotrophic” or “neuroprotective.” Seventeen out of 26 selected neurotrophic genes and 19 out of 35 selected neuroprotective genes are highly expressed in all cell samples (“highly” here means above the average expression level of all sequences printed on the arrays) (Fig. 5a, b). With a few exceptions, however, the intraocular MGCs manifested an overall higher expression than the other cell types (Fig. 5a–c). MGCs are the major supportive and protective neuroglial cells in the neuroretina, and the higher expression of neurotrophic and neuroprotective mRNAs is in agreement with the above cell transplant experiment results in which MGCs were more beneficial than other primary differentiated cells in terms of rescuing degenerating retinal vision (Fig. 4b). Although MGCs express higher amounts of neurotrophic and neuroprotective transcripts than SDSCs, they were less effective at rescuing degenerating retinas than SDSCs (Figs. 2e and 4b), suggesting that SDSCs might secrete a specific paracrine factor(s) that more effectively protects and/or nurtures the remaining photoreceptor cells. SDSCs were the best candidate for rescuing the degenerating retinas upon transplantation, but the gene expression levels of the selected neurotrophic and neuroprotective factors were overall lowest among the tested cells. This suggests that it is probably not the amount of the factor(s) but the duration of time that these factors are present in the “neighborhood” that determines the extent to which the transplanted cells could positively affect the nearby neurons. In addition, we also selected some growth factors that might be beneficial for neuron survival. Thirteen growth factors, including *Ctgf*, *Hdgf*, *Hgf*, *Pdgfa/b/c/d*, and *Tgfb2/3*, in addition to other known neuroprotective growth factors, such as *Fgf*, *Igf1/2*, and *Vegfa/b/c*, were highly expressed in selected cells (Fig. 5c). In contrast, more popular neuroprotective growth factors, such as *Fgf*, *Hgf*, *Ngf*, *Bdnf*, *Cntf*, and *Gdnf*, were significantly underexpressed (Fig. 5c).

### Detection of factors that cells secreted into the cell culture media

**Fig. 6:**
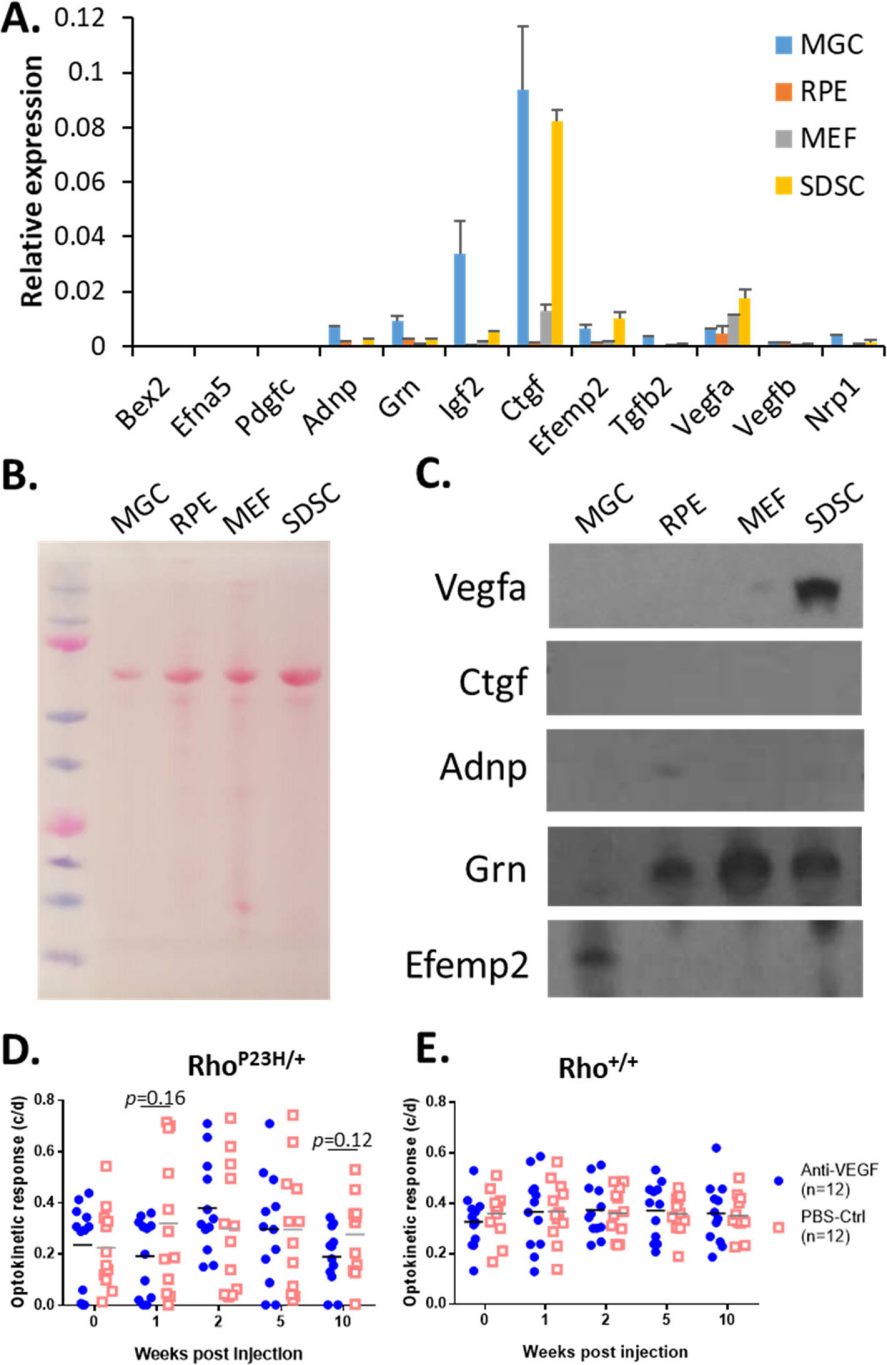
Identification of paracrine factors that nurture and/or protect retinal neurons. **a** Based on Affymetrix microarray gene expression data, nine out of 12 genes selected for qPCR were confirmed to be expressed in the cultured cells. **b** Total proteins were isolated from cell-conditioned media, separated by SDS-PAGE, transferred onto a PVDF membrane, and stained with 0.5% (w/v) Ponceau S. **c** Among the 5 selected genes [27–32], three encoded proteins were detected by WB to be present in the cell-conditioned media. **d** The neuroprotective effect of Vegfa was evaluated at the indicated time points by OKR after an intravitreal injection of the anti-VEGF antibody (bevacizumab) into Rho^P23H/+^ and **e** Rho^+/+^ mice at P40

The gene expression data suggest that some proteins encoded by the selected neurotrophic and neuroprotective genes might have been produced in cells and secreted into the media when cultured in vitro or into the subretinal space when transplanted in vivo. To confirm the microarray data, we selected 12 genes for further qPCR analysis based on three criteria: higher microarray expression, known ocular defects caused by their genetic mutations, and/or presence in the eye (Fig. 5, red-letter genes). Nine out of the 12 genes were confirmed by qPCR to be present in our cell samples (Fig. 6a). To detect if the proteins encoded by the confirmed genes are present in the cell culture media, we harvested 2-day media of confluent cultures and concentrated the proteins in the media using the trichloroacetic acid (TCA) precipitation protocol [23]. The concentrated protein samples were thereafter electrophoresed by SDS-PAGE for WB analysis [22]. As a result, three out of 5 selected extracellular secretory factors (Vegfa [27–29], Ctgf [30], Adnp [31], Grn [32], and Efemp2), i.e., Vegfa, Grn, and Efemp2, were detected by WB (Fig. 6b, c).

### Intraocular neutralization of Vegfa enhances the retinal degeneration of Rho^P23H/+^ mice

Among the above factors detected in the cell-conditioned media, Vegfa and Efemp2 were secreted by SDSCs and MGCs, respectively, while Grn was secreted by all cells but MGCs (Fig. 6c). As demonstrated above, SDSCs had the best capacity to retain functional photoreceptors in Rho^P23H/+^ mice (Fig. 2e). Vegfa was the sole detected cytokine secreted by SDSCs among the tested cells, thereby providing a better candidate for further neuroprotection functional analysis. VEGFA is the most important cytokine that induces angiogenesis and neovascularization, and neutralization of VEGFA is a primary treatment for neovascular AMD and diabetic retinopathy (DR) patients [33, 34]; but it is also a known neuroprotective cytokine [35]. Therefore, we reasoned that neutralization of VEGFA not only stops or even regresses neovascularization but also diminishes its neuroprotective property, thereby causing adverse effects on the neuroretina. Several pieces of evidence have shown that VEGFA could protect retinal ganglion cells (RGCs) [27–29], but how it affects photoreceptors is still an open question. Therefore, we selected Vegfa for further loss-of-function analyses. We investigated the adverse effect of an intravitreal injection of the anti-VEGF antibody (bevacizumab, also known as Avastin, 75 μg/eye anti-VEGF_164_) on the visual function of Rho^P23H/+^ and Rho^+/+^ mice at P40. Compared to the PBS control, anti-VEGF-treated eyes manifested lower OKR values in Rho^P23H/+^ mice, particularly at the first and last week after the intravitreal injection of the anti-VEGF antibody, though not statistically significant (Fig. 6d). In contrast, little change in OKR between the anti-VEGF-treated and PBS control eyes was detected in Rho^+/+^ wild-type mice (Fig. 6e). Taken together, we conclude that VEGF has some neurotrophic and neuroprotective effects, particularly in retinal degenerating eyes, but the adverse effect of anti-VEGF application on healthy murine eyes appears minimal, at least in the short term (within 10 weeks).

## Discussion

### The therapeutic effects of transplanted cells on the remediation of retinal degeneration depend on the cell survival duration

We have developed a cell sphere-induced reprogramming biotechnology to reset a terminally differentiated lineage back to a more immature status with higher capacities to proliferate and to transdifferentiate [17]. RPE cell-derived SDSCs are immortal and have the capacity to differentiate into cells that express neural markers in vitro (Fig. 1b–d). Our transplantation experiments also indicated that SDSCs could spread from injection sites and possibly integrate into the retina in vivo, where some of them expressed the neural marker recoverin (Rec) and synthesized the pigment in vivo (Fig. 3). This higher potential of SDSCs distinguishes them from the terminally differentiated primary lineages, i.e., MEF, MGC, and RPE cells, that usually only moved into the compatible tissue of the same embryonic origin (Fig. 4e–g). SDSCs have shown the best capacity, among the other lineages used in the experiments, to remediate retinal degeneration in Rho^P23H/+^ mice by retaining more ONL nuclei and longer OS (Figs. 2e and 4a, b). SDSCs have also shown a higher proliferative capacity in culture (Fig. 4c) and are seemingly more survivable than the other lineages used after transplantation to recipients (Fig. 4h) and thereby can continue to secrete beneficial factors to remediate degenerating retinas for a longer time. Unfortunately, we did not extend our transplant experiments to determine exactly how long SDSCs could survive in the subretinal space. In addition to SDSC, MGC was the best survivable cell type with a higher proliferation rate among the primary cell types (Fig. 4c) and therefore had a comparable effect on rescuing the OKR visual capacity compared to SDSC (Figs. 2e and 4b). Other differentiated cell populations survived relatively shorter than 4 weeks (Fig. 4e–g), and their positive effects on retaining the OKR of Rho^P23H/+^ mice started to drop at 2 weeks post transplantation (Fig. 4b). This shorter existence of transplanted cells in the subretinal space is likely due to the immune rejection response of the incompatible recipient animals because the cell transplantation procedure disrupts the blood barrier of the ocular immune privilege confinement. Based on our observation, adult stem cells with capacities to live longer in recipients would serve better as a paracrine factor provider to remediate degenerating retinas than those terminally differentiated cell types for cell-based therapies.

### Functional integration of transplanted cells is the key for cell replacement therapies but is not necessarily required for RP remediation

The initial idea for cell-based therapies was to functionally replace defective/diseased target cells in affected tissues, particularly stem cells such as the limbal stem cell that would continue to provide needed lineages in the cornea for cellular homeostasis and damage repair [2]. However, this idea does not work when such a stem cell population is missing or their migration in situ is very limited, such as in mammalian central nerve tissues [14]. In such cases, a fully differentiated lineage such as photoreceptors or RPE cells can be grafted to substitute for lost cells in their compatible tissue where they need to re-establish a new partnership with existing lineages [36, 37]. A large number of experiments with pluripotent stem cell (PSC)-derived target lineages have been conducted and have shown promising but limited potential [36]. The major problem is the limited functional integration not only in the retina but also in other tissues such as the cardiac muscle [38]. The functional improvement after cell transplantation has been largely attributed to the paracrine effect [2, 38]. This conclusion is mostly based on observations in which very few transplanted cells survived for long and the visual improvement was not sustained accordingly. If the paracrine factor(s) secreted by grafted cells is the true cause for the improvement of diseased tissues, it is logical to think about formulating a relatively easy and direct strategy to administer the paracrine factor(s) either systematically through the circulation or locally by targeted delivery. Many extracellular factors and cytokines have been indicated to nurture and/or protect retinal neurons, but which of them are possibly secreted by transplanted cells has yet to be identified. Our experiments were designed to clarify the effects of grafted cells (functional integration vs. paracrine remediation) and to identify the major paracrine factors that are beneficial for remediation of retinal degeneration.

### Vegfa and other cell transplantation-induced factors like Grp78 are the potential factors that benefit the survival of both transplanted cells and degenerating retinal cells

Our strategy in the search for major secretory neurotrophic and neuroprotective factors in the remediation of retinal degeneration was initially based on gene expression data and then on the actual presence of factors in the cell culture media. The identified factor Vegfa was further confirmed by a loss-of-function approach using anti-VEGF antibody. In our experiment, Vegfa was identified as a retinal neuroprotective factor that is important for nurturing and maintaining photoreceptors in vivo as reported [35, 39]. The utilization of anti-VEGF medications for treating ocular neovascularization that would increase visual correction in the short term might actually result in long-term damage to retinal neurons. Unfortunately, our anti-VEGF animal experiment lasted only 10 weeks, although the adverse effect of the one-time anti-VEGF antibody application on the OKR visual response in Rho^P23H/+^ mice was clearly detected right after the application and at 10 weeks (Fig. 6d). More experiments with neovascularized animals, such as mouse model of alkali-induced corneal neovascularization, and multiple applications of anti-VEGF for a much longer duration may be needed to further validate the results. We therefore need to carefully evaluate such adverse effects in patients treated with long-term anti-VEGF drugs and search for new medications that not only block ocular neovascularization but also have little adverse effects on healthy retinal neurons. It has been known that some stress-induced factors like Grp78 facilitate the survival of degenerating photoreceptor cells [40]. To test whether cell transplantation would also induce the expression of Grp78 in the grafted retina, we immunostained the cryosections of both PBS control and SDSC-transplanted Rho^P23H/+^ retinas with and without the GRP78 antibody. Indeed, the SDSC-transplanted retinal cells did show a higher expression of Grp78 (Fig. S1A) than the PBS control (Fig. S1B), suggesting that the subretinal transplantation of SDSCs may cause some stress response in the retina, which in turn benefits the survival of degenerating photoreceptor cells [40, 41]. It appears that the transplanted cells and/or the stressed retinal cells secrete paracrine and/or autocrine factors like Vegf that bind to their according receptors like Vegfr and Grp78 initiating signals to activate pathways through activation of Akt leading to the survival of the transplanted cells and/or remaining photoreceptor cells of the degenerating retina though the actual ligand(s) for Grp78 is not clear (Fig. 4i). In fact, GRP78 is also an important cytoplasmic protein in activation of Akt-associated cell survival pathway through activating BCL-2 and inhibiting CASP12 activities (Fig. 4i) [40, 41].

## Conclusions

The cellular therapy-produced benefits in remediating retinal degeneration are mostly, if not completely, due to a paracrine effect of implanted cells on the remaining retinal neurons.

## Supplementary information

**Additional file 1:Supplementary Figure S1 (A).** A higher expression of the tress-induced GrP78 in the retina with the transplanted cells compared to **(B)** the PBS control retina in the Rho^P23H/+^ mice 4 weeks after a subretinal transplantation of SDSCs at P40 suggests that Grp78 participates in the survival of both the transplanted and photoreceptor cells in the degenerating retina.

**Additional file 2:Supplementary Table 1.** Primary antibodies used for Western Blot (WB), Immunohistochemistry (IHC) and immunofluorecscne (IF).

**Additional file 3:Supplementary Table S2.** Primers used for real-time PCR detection.

## Data Availability

Please contact the author for data requests.

## Abbreviations

AMD: Age-related macular degeneration
DR: Diabetic retinopathy
ESC: Embryonic stem cell
GCL: Ganglion cell layer
IPL: Inner plexiform layer
INL: Inner nuclear layer
iPSC: Induced pluripotent stem cell
IS: Inner segment
MEF: Mouse embryonic fibroblast
MGC: Müller glial cell
OKR: Optokinetic response
ONL: Outer nuclear layer
OPL: Outer plexiform layer
OS: Outer segment
RP: Retinitis pigmentosa
PSC: Pluripotent stem cell
qPCR: Quantitative real-time PCR
Rec: Recoverin
RGC: Retinal ganglion cell
Rho: Rhodopsin
RPE: Retinal pigment epithelium
SDSC: Sphere-induced stem cell
WB: Western blotting
