# Supplementary figures and images for: Paracrine effects of intraocularly implanted cells on degenerating retinas in mice
Source: Stem Cell Res Ther. 2020 Mar 31;11:142. doi: 10.1186/s13287-020-01651-5 (PMC7326149; doi:10.1186/s13287-020-01651-5)

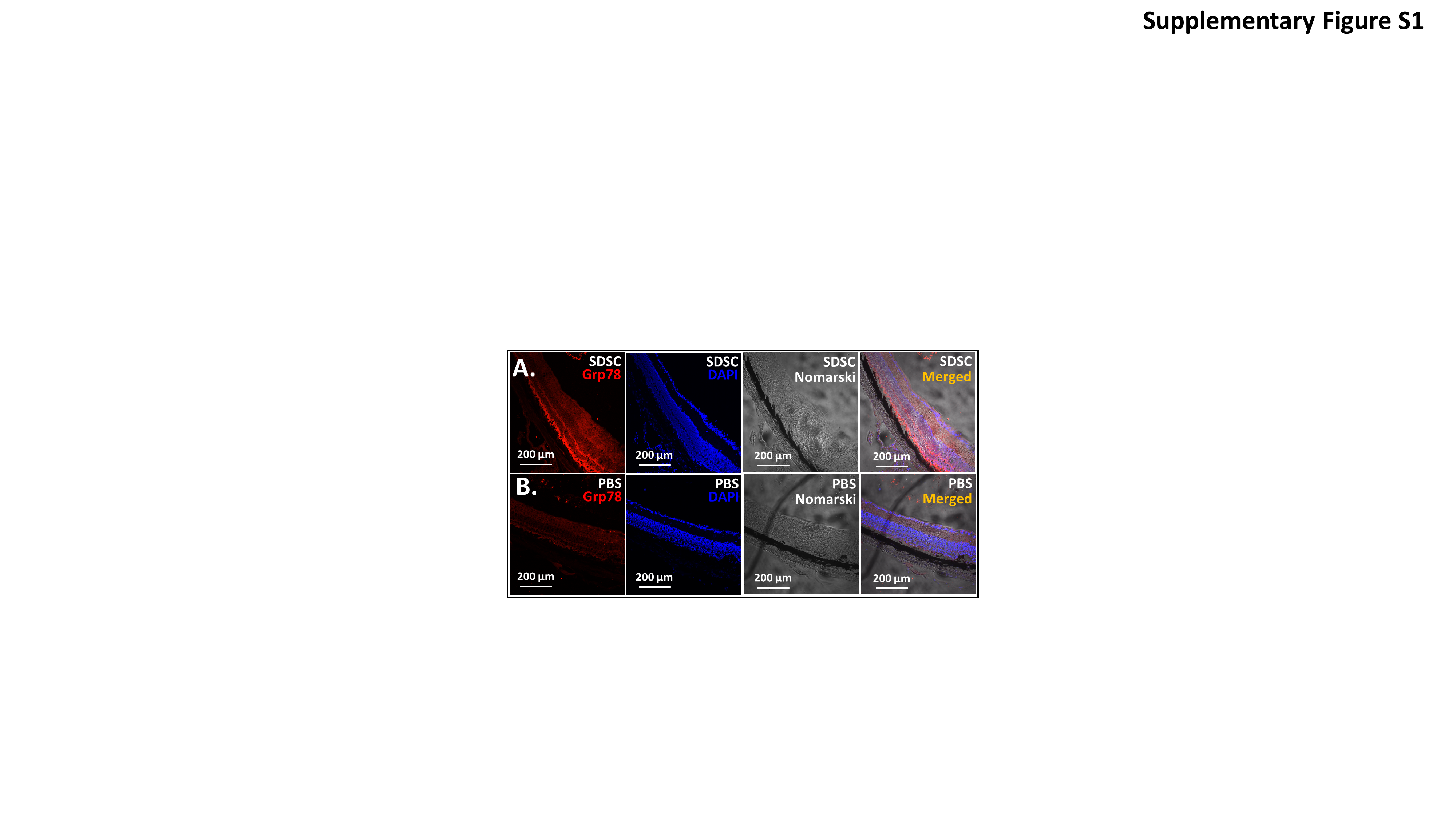

Supplement: Supplementary file 1 — Additional file 1:Supplementary Figure S1 (A). A higher expression of the tress-induced GrP78 in the retina with the transplanted cells compared to (B) the PBS control retina in the RhoP23H/+ mice 4 weeks after a subretinal transplantation of SDSCs at P40 suggests that Grp78 participates in the survival of both the transplanted and photoreceptor cells in the degenerating retina. [file 13287_2020_1651_MOESM1_ESM.tif]
