# Supplementary material for: Paracrine effects of intraocularly implanted cells on degenerating retinas in mice
Source: Stem Cell Res Ther. 2020 Mar 31;11:142. doi: 10.1186/s13287-020-01651-5 (PMC7326149; doi:10.1186/s13287-020-01651-5)
Supplement: Supplementary file 2 — Additional file 2:Supplementary Table 1. Primary antibodies used for Western Blot (WB), Immunohistochemistry (IHC) and immunofluorecscne (IF). [file 13287_2020_1651_MOESM2_ESM.docx]

**Supplementary Table 1.** Primary antibodies used for Western Blot (WB), Immunohistochemistry (IHC) and immunofluorecscne (IF)

| **Antibody name** | **Manufacturer** | **Catalog #** | **Application** | **Dilution** |
| --- | --- | --- | --- | --- |
| Actb | Sigma | a5441 | WB | 1:1000 |
| Anti-mouse IgG-Alexa fluor 488 | Invitrogen | A11008 | IHC, IF | 1:500 |
| Anti-rabbit IgG-Alexa fluor 568 | Invitrogen | A11036 | IHC, IF | 1:500 |
| Recoverin | Millipore | AB5585 | IHC, IF | 1:100 |
| Rhodopsin | Millipore | MAB5356 | IHC, IF | 1:200 |
| Erk | Santa Cruz | sc-154 | WB | 1:500 |
| p-Erk | Cell Signaling | 9101S | WB | 1:50 |
| Akt | Santa Cruz | sc-8312 | WB | 1:500 |
| p-Akt | Santa Cruz | sc-33437 | WB | 1:200 |
| Vegfa | Cell Signaling | 4912 | WB | 1:500 |
| Grn | R&D | AF25571 | WB | 1:1000 |
| Efemp2 | ProteinTech | 12004 | WB | 1:1000 |
| Ctgf | R&D | MAB91901 | WB | 1:1000 |
| M-csf | R&D | MAB4161 | WB | 1:1000 |
| Adnp | R&D | MAB5919 | WB | 1:1000 |
| Grp78 | Santa Cruz | sc-13539 | IHC | 1:100 |
