# Supplementary material for: Paracrine effects of intraocularly implanted cells on degenerating retinas in mice
Source: Stem Cell Res Ther. 2020 Mar 31;11:142. doi: 10.1186/s13287-020-01651-5 (PMC7326149; doi:10.1186/s13287-020-01651-5)
Supplement: Supplementary file 3 — Additional file 3:Supplementary Table S2. Primers used for real-time PCR detection. [file 13287_2020_1651_MOESM3_ESM.docx]

**Supplementary Table S2.** Primers used for real-time PCR detection

| **Primer name** | **Sequence (5'—3')** | **Tm ˚C** | **Amplicon (bp)** |
| --- | --- | --- | --- |
| Mm Adnp LP | GCGTTCACTTCGAAAAGGAG | 54.1 | 171 |
| Mm Adnp RP | AACGGCAATACGGACAAGAC | 55.4 |  |
| Mm Bex2 LP | GAAGAAAAGCCACAGGATGC | 54.3 | 212 |
| Mm Bex2 RP | TGTCTCACATCATCCCCAAA | 53.7 |  |
| Mm Ctgf LP | CAAAGCAGCTGCAAATACCA | 55.6 | 220 |
| Mm Ctgf RP | GGCCAAATGTGTCTTCCAGT | 55 |  |
| Mm Efemp2 LP | GCCCAAACCTGTGTCAACTT | 56 | 250 |
| Mm Efemp2 RP | AACGGATCTGAAAGGCATTG | 53.3 |  |
| Mm Efna5 LP | GCTGCTCTTTCTGGTGCTCT | 57.6 | 165 |
| Mm Efna5 RP | GGGCAGAAAACATCCAGGTA | 54.8 |  |
| Mm Grn LP | ACCCTCTTCTGGACACATGG | 56.6 | 158 |
| Mm Grn RP | GCCATCACCACAAGACACAC | 56.5 |  |
| Mm Igf2 LP | GTCGATGTTGGTGCTTCTCA | 55.1 | 195 |
| Mm Igf2 RP | AAGCAGCACTCTTCCACGAT | 56.8 |  |
| Mm Nrp1 LP | GGAGCTACTGGGCTGTGAAG | 57.6 | 208 |
| Mm Nrp1 RP | ACCGTATGTCGGGAACTCTG | 56.6 |  |
| Mm Pdgfc LP | AGTCCAACCTGAGCAGCAAGT | 59 | 166 |
| Mm Pdgfc RP | ACTAATCTCCACACCAGCACCA | 58.2 |  |
| Mm Vegfa LP | CACGACAGAAGGAGAGCAGA | 56.4 | 190 |
| Mm Vegfa RP | CAGGGCTTCATCGTTACAGC | 56.1 |  |
| Mm Vegfb LP | CCTGACGATGGCCTGGAATGT | 59.6 | 163 |
| Mm Vegfb RP | TGTCTGGCTTCACAGCACTCTC | 59.2 |  |
| Mm Tgfb2 LP | CAGCGCTACATCGATAGCAA | 55 | 199 |
| Mm Tgfb2 RP | CCTCGAGCTCTTCGCTTTTA | 54.6 |  |
| Mm Gapdh LP | AACGACCCCTTCATTGAC | 56.0 | 191 |
| Mm Gapdh RP | TCCACGACATACTCAGCAC | 56.0 |  |
